# Case of Injury of the Head from a Fall, Causing Concussion of the Brain, without the Usual Symptoms

**Published:** 1844-11-16

**Authors:** Charles Huston


### THE MEDICAL EXAMINER,

### And Record of Medical Science.

Vol. VII.] PHILADELPHIA, SATURDAY, NOVEMBER 16, 1844. [No. 23.

### CASE OF INJURY OF THE HEAD FROM A FALL, CAUSING CONCUSSION OF THE BRAIN, WITHOUT THE USUAL SYMPTOMS.

BY CHARLES HUSTON, M.D.

Daniel Smith, aged 10 years, fell, on the 6th of September last, from a height of about fifteen feet, and struck his head a little below and behind the parietal protuberance, on a marble step. I was called to him twenty hours after the occurrence, and found him with a pulse of 48, having had severe headache and occasional attacks of vomiting since the accident:—his mind was not affected, and he answered intelligibly all my questions relating to his injury. On examining the head I found ecchymosis to considerable extent, but could form no decided opinion as to the state of the bone. Ordered twenty leeches to the injured part, magnesiæ sulphas to open his bowels, and low diet.

7th. Bowels opened once; pulse the same; pain in the head relieved, but great intolerance of light and sound. In the afternoon, Prof. Pancoast was called in, the symptoms remaining the same; ordered hydrarg. chlorid. mitis, gr. v., the head to be shaved, and cloths wrung out of cold water applied to it.

8th. Pulse 54; bowels regular; intolerance of light and sound still remaining.

The symptoms continued very much the same until the 12th, the pain in the head coming on in paroxysms, but with lengthened intervals, and the pulse varying from 50 to 60; on this day he had a slight convulsion, with a wild expression of countenance and dilated pupils; the pulse still 60, and of the natural force: ordered mustard foot-bath and a purge. Nothing of the kind occurred afterwards, the pulse gradually rose to 70 but no higher, the pain in the head ceased, and on the 17th I discharged him convalescent.

Was this a case of concussion or compression? Had it been the former, we ought to have had reaction, with fever, &c.; and on the other hand, if it was compression we should have witnessed somnolency, diminished sensibility of the nervous system, &c., none of which existed. A *palliative treatment* was that which appeared to me to be indicated, and I am happy to be able to say, it was attended with the most beneficial results.

